# The effect of length of birth interval on the risk of breast cancer by subtype in grand multiparous women

**DOI:** 10.1186/s12885-019-5404-z

**Published:** 2019-03-04

**Authors:** Sushmita Katuwal, Juha S. Tapanainen, Eero Pukkala, Antti Kauppila

**Affiliations:** 1 Faculty of Social Sciences, University of Tampere, Kauppi Campus, Arvo Ylpön Street 34, 33520 Tampere, Finland; 2 Department of Obstetrics and Gynecology, University of Helsinki, Helsinki University Hospital, Helsinki, Finland; 3 Department of Obstetrics and Gynecology, Oulu University Hospital, University of Oulu, Medical Research Center Oulu, Oulu, Finland; 4 Department of Obstetrics and Gynecology, University of Oulu and Oulu University Hospital, Medical Research Center, PEDEGO Research Unit, Oulu, Finland; 5 Finnish Cancer Registry, Institute for Statistical and Epidemiological Cancer Research, Helsinki, Finland

**Keywords:** Breast cancer, Aetiology, Risk factor, Birth spacing, Lobular, Ductal, Age at diagnosis, Menopause, Pregnancy, Age at first birth, Age at last birth, birth interval

## Abstract

**Background:**

The length of interval between successive childbirths (birth interval) might influence the incidence of breast cancer, either by stimulating or by inhibiting the factors that are responsible for the initiation of breast cancer or its early development.

**Methods:**

This is a case-control study nested in a cohort of 47,479 Finnish grand-multiparous (GM) women born after 1934, and registered as having had at least five births before 2013. The 1354 women with breast cancer diagnosis were compared with controls (1:5) matched by parity and date of birth of the mother. Conditional logistic regression was used to estimate odds ratios of the risk of ductal and lobular breast cancer subtypes associated with each of the intervals between the 1st and 5th birth, stratified by age at diagnosis. Age at first and last birth before index date were used as covariates.

**Results:**

Increased intervals between the 1st and 5th births were associated with an increased risk of lobular breast cancer. In contrast, regarding ductal cancer, premenopausal women with shorter length of interval (< 2 years) between the 1st and 2nd birth had greater risk and longer intervals (3+ years) were associated with reduced risk. Spacing between the 2nd and 5th birth did not influence the risk of ductal breast cancer.

**Conclusion:**

The findings of our study suggest that the effect of the length of birth interval on breast cancer depends on the age and histology. The protective effect of shorter birth intervals on lobular breast among postmenopausal women and the opposite effect on ductal cancer in premenopausal women may reflect distinct differentiation and functional roles of lobular and ductal cells, and possibly also different response to hormonal exposure.

## Background

Age at first birth and the number of pregnancies are well-known risk factors of breast cancer [1–6], and each pregnancy after the first one induces an additional long-lasting risk reduction until the eighth pregnancy (6). However, pregnancies have dual effects on breast cancer: the long-lasting protective effect of a pregnancy at a young age is preceded by a transient increase in risk (up to 3–10 years) after the birth [2, 4–7]. Possibly, a growth-stimulating effect of pregnancy steroids on silent subclinical breast cancer explains this kind of dual effect of pregnancy on breast cancer.

Reproductive events and their timing can affect breast tissue differentiation through hormonal mechanisms [8–11]. Levels of estrogenic hormones, which are important for development and maturation of the fetus, increase steadily during pregnancy and reach a peak in the third trimester. Estrogens play several roles in neoplastic transformation of breast tissue, either as carcinogenic agents or as permissive, promotional and tumor growth-inducing agents [12, 13]. Birth interval (the time between two successive childbirths) seems to be connected to the early phases of breast cancer development [5, 6, 9]. Shorter intervals between births, particularly between the 1st and 2nd birth, lower the risk of breast cancer [14–19]. The interval between the 1st and 2nd pregnancy may influence breast cancer development, either by stimulating or by inhibiting the factors that are responsible for the initiation of breast cancer or its early growth. An earlier study among Finnish grand multiparous women indicated the natural inter-birth interval of these women is about 1.5 years [5]. We therefore hypothesize that the “natural birth interval” of 1.5 years would carry the lowest risk of breast cancer among grand multiparous women.

Studies on pregnancy-related risk factors including birth intervals have mostly covered women with just a few or only one childbirth. There is an evident need for further studies on the importance of the 1st birth interval, and also later intervals on the etiology of specific subtypes of breast cancer extended to women with higher parity. Also, the consideration of age, stage and characteristics of the tumor at diagnosis is essential for clear understanding of the association between birth intervals and breast cancer risk. The present population-based study was especially aimed to clarify the role of each birth interval between the 1st and 5th birth as separate risk factors in ductal and lobular breast cancer subtypes among grand-multiparous (GM) women.

## Methods

This is a retrospective case-control study nested in a Finnish population-based cohort of GM women. The cohort, obtained from the Finnish National Population Register (NPR), consists of 47,479 Finnish women who were born after 1934, and registered as having had at least five births before January 1st, 2013. The NPR contains links between mothers and their children born after September 1953.

The first breast-cancer cases (ICD-10 C50) diagnosed between the 5th childbirth and the 31st of December 2014 in GM women were included in the study. Altogether, 1354 such cases were identified in the Finnish Cancer Registry, in an automatic record linkage from NPR via unique personal identity codes assigned to each resident of Finland. The cancers recorded at the Finnish Cancer Registry have been notified by hospitals, pathological and hematological laboratories, physicians and dentists, and from death certificates [20]. For this study, we also extracted data from the Finnish Cancer Registry on histological type and spread of the disease at the time of diagnosis (localized, advanced) which is coded according to the ICD-O-3 system. The NPR, includes accurate information on a very high proportion of childbirths of women born after the mid-1930s and the Finnish Cancer Registry is virtually complete as regard to cancer incidence since 1953 [20, 21].

For each case, five controls were randomly selected among GM women cohort who were at the risk of breast cancer at the time of cancer onset of case and fulfilled the matching criteria. Matching was done by date of birth of mother and number of children and tolerance of ±6 months was allowed for the date of birth. Women who had emigrated or died before the index date were not eligible as controls.

A conditional logistic regression model for matched case-control data was used for both univariate and multivariate analyses to examine the associations between study variables and breast cancer. The variables included in the model were each of the four birth intervals until the fifth birth, with age at first birth and age at last birth as covariates. Odds ratios (ORs) with 95% confidence intervals (CIs) were calculated to evaluate the associations between the predictor variables and breast cancer.

We categorized the lengths of the first four birth intervals into four categories: < 1 year, 1–1.99 years (reference category), 2–2.99 years and 3+ years. Longest interval category is 3+ years. Age at first birth is categorized into four categories: < 20 years (reference category), 20–24 years, 25–29 years and 30+ years. Because age at last birth might not be a fully independent risk factor but depends on the lengths of birth intervals, we performed the analyses in two models, with and without the age at last birth before index date. The findings of both models were virtually identical and we therefore presented only results from the model including age at last birth in the model, categorized into four categories: < 30 years (reference category), 30–34 years, 35–39 years and 40+ years. In addition, the logistic regression model was also adjusted for average interval between 5th and the last birth before index date. Potential interactions between the study variables were evaluated by adding the interaction terms in the logistic regression model.

Since some previous studies on birth intervals and breast cancer risk have been based on average interval between first and last birth, we made an alternative analysis based on models where the four separate intervals between the 1st and 5th birth were replaced by their average. In this analysis, the average interval was categorized to < 2 years (reference category), 2–2.99 years and 3+ years.

Ductal and lobular subtypes of breast cancer were analyzed separately and also stratified according to the spread of the disease at the time of diagnosis (localized and advanced). Further stratification was carried out for age at diagnosis (< 50 years, later called “premenopausal”, and ≥ 50 years, “postmenopausal”). Since there were only 32 cases of lobular breast cancer diagnosed at ages of < 50 years, no separate analysis was carried out for this group. All analyses were performed using R statistical software, version 3.2.3.

## Results

Overall, 76% of women with ductal breast cancer subtype and 86% of women with lobular breast cancer subtype in our study were diagnosed at the age 50 years or above (Table 1). The women with different breast cancer subtypes were similar in terms of average age at 1st birth (22.0 years for ductal breast cancer group and 22.8 years in the lobular breast cancer group), and age at 5th birth (33.6 and 34.8 years for ductal and lobular breast cancer groups, respectively). The respective mean ages at cancer diagnosis were also similar in these groups (58.4 years for ductal and 58.9 years for lobular breast cancer groups).

**Table 1:** Number (N) and percentage (%) of women with first breast cancer diagnosed after 5th birth (1974–2014) among 47,479 women in Finland born after 1935 and registered to have at least five biological children, by histology, stage of cancer, number of children (parity) and age at breast cancer diagnosis

Variable	Age at breast cancer diagnosis						Percentage of breast cancers diagnosed in age 50+ out of all breast cancers
	All ages		< 50 years		50+ years		
	N	%	N	%	N	%	
All	1354	100	312	100	1042	100	77
Histology & stage							
Ductal	1037	77	252	81	785	75	76
Localized	537	52	95	38	442	56	82
Advanced	434	42	142	56	292	37	67
Unknown	66	6	15	6	51	7	77
Lobular	207	15	32	10	175	17	86
Localized	79	38	8	25	71	41	90
Advanced	108	52	23	72	85	49	79
Unknown	20	10	1	3	19	10	95
Other/unknown	110	8	28	9	82	8	75
Parity							
5	857	63	179	58	678	65	79
6	264	20	60	19	204	20	77
7	93	7	29	9	64	6	69
8+	140	10	44	14	96	9	69

Table 2 provides a comparison of the effect of the effect of average interval length between the

**Table 2:** Multivariate conditional logistic regression analysis for the average intervals between 1st and 5th birth as predictors of breast cancer, ductal breast cancer (< 50 years and 50+ years) and lobular breast cancer (50+ years) risk among multiparous women in Finland. Reference: Average interval < 2 years

	Average interval between 1st and 5th birth			
Variable	2–2.99 years		3+ years	
	OR	95% CI	OR	95% CI
All breast cancer	0.95	0.79–1.14	1.15	0.89–1.47
Ductal breast cancer	0.87	0.70–1.07	1.02	0.77–1.35
Ductal (< 50 years)	0.87	0.41–1.85	0.36	0.13–1.03
Ductal (50 + years)	0.93	0.73–1.18	1.22	0.88–1.71
Lobular breast cancer	2.21	1.28–3.79	2.98	1.47–6.03
Lobular (50+ years)	2.24	1.24–4.01	2.65	1.23–5.70

*OR* odds ratio, *95% CI* 95% confidence interval

Models were adjusted for average interval between 5th and the latest birth before index date, age at first and last birth

1st and 5th births by breast cancer subtypes (ductal and lobular) and age at diagnosis (> 50 years and 50+ years). For all breast cancers combined and for all ductal cancers combined, the length of the interval was not associated with risk of breast cancer. However, differences were seen for lobular cancer (both in total and in those diagnosed at 50+) with significantly higher OR’s associated with longer birth intervals (2–2.99 years and 3+ years vs < 2 years). In contrast, for ductal cancer diagnosed under 50, longer intervals, especially of 3+ years, were associated with reduced risk.

While examining the difference between specific intervals it was observed that for ductal cancer at < 50 years the longer intervals were protective only between the 1st and 2nd and 2nd and 3rd intervals (Table 3). Especially, short intervals (< 1 year) were associated with significantly higher risk in these younger ductal cancer cases between the 1st and 2nd and 3rd and 4th intervals. For the lobular cancers diagnosed at 50+ years, the longer intervals were associated with increased risk between each of the intervals and the ORs was significant for 3+ years.

**Table 3:** Multivariate conditional logistic regression analysis for the Intervals between births, age at first birth and age at last birth as predictors of ductal breast cancer diagnosed at the ages of < 50 years and 50+ years, and lobular breast cancer diagnosed at the age of 50+ years

Variable	Ductal (< 50 years)			Ductal (50+ years)			Lobular (50+ years)		
	N	OR	95% CI	N	OR	95% CI	N	OR	95% CI
Intervals between births									
1st and 2nd									
< 1 year	18	3.56	1.06–11.95	39	1.00	0.69–1.43	8	0.78	0.35–1.76
1–1.99 years	136	1.00	Ref.	456	1.00	Ref.	102	1.00	Ref.
2–2.99 years	62	0.86	0.44–1.70	138	0.83	0.67–1.03	33	1.01	0.64–1.60
3+ years	36	0.38	0.14–1.00	152	1.23	0.98–1.54	32	1.45	0.88–2.42
p-trend			< 0.001			0.47			0.18
2nd and 3rd									
< 1 year	9	0.43	0.07–2.50	42	1.06	0.74–1.52	6	0.70	0.28–1.75
1–1.99 years	123	1.00	Ref.	363	1.00	Ref.	68	1.00	Ref.
2–2.99 years	48	0.39	0.18–0.83	162	0.90	0.73–1.11	45	1.46	0.94–2.28
3+ years	72	0.50	0.23–1.11	218	0.98	0.79–1.20	56	1.34	0.84–2.13
p-trend			0.91			0.55			0.11
3rd and 4th									
< 1 year	11	6.76	1.52–30.11	25	0.80	0.51–1.25	9	2.07	0.89–4.79
1–1.99 years	83	1.00	Ref.	303	1.00	Ref.	52	1.00	Ref.
2–2.99 years	60	1.32	0.60–2.90	183	1.04	0.84–1.28	38	1.35	0.82–2.22
3+ years	98	1.36	0.65–2.88	274	0.90	0.74–1.11	76	1.64	1.01–2.65
p-trend			0.07			0.55			0.75
4th and 5th									
< 1 year	10	0.54	0.10–3.00	42	1.19	0.81–1.74	8	0.97	0.28–1.59
1–1.99 years	72	1.00	Ref.	218	1.00	Ref.	43	1.00	Ref.
2–2.99 years	60	1.13	0.54–2.35	167	1.07	0.85–1.33	39	1.43	0.85–2.38
3+ years	110	0.88	0.41–1.89	358	1.00	0.80–1.23	85	1.43	0.87–2.35
p-trend			0.45			0.43			0.26
Age at first birth									
< 20 years	66	1.00	Ref.	275	1.00	Ref	48	1.00	Ref.
20–24 years	105	1.02	0.49–2.14	339	0.89	0.74–1.08	72	1.00	0.65–1.53
25–29 years	64	1.36	0.55–3.31	143	1.16	0.88–1.51	39	1.82	1.02–3.29
30+ years	17	2.65	0.57–12.22	28	1.19	0.72–1.95	16	5.35	2.12–13.47
p-trend			0.12			0.33			< 0.001
Age at last birth									
< 30 years	15	1.00	Ref.	140	1.00	Ref.	17	1.00	Ref.
30–34 years	50	2.30	0.63–8.40	192	1.17	0.89–1.53	43	1.98	1.02–3.88
35–39 years	90	4.13	1.02–16.59	181	1.06	0.77–1.46	41	1.44	0.66–3.16
40+ years	97	5.63	1.41–22.55	272	1.17	0.83–1.65	74	1.89	0.81–4.44
p-trend			0.03			0.51			0.25

*N* number of cancer cases, *OR* odds ratio, *95% CI* 95% confidence interval

Models were adjusted for average interval between 5th and the latest birth before index date

Increasing age at first and last birth, included in the models, were both associated with increased breast cancer risk among both subtypes and age groups (Table 3). Significantly increased ORs were observed for age at last birth at 35+ years (vs. < 30 years) for ductal cases diagnosed under 50 (OR 5.63, 95% CI 1.41–22.55). For age at first birth, significantly increased risk was observed at age of 30+ years (vs. < 20 years) for lobular cancer diagnosed at 50+ (OR 5.35, 95% CI 2.12–13.47).

There were no statistically significant interactions between the birth intervals and age at first birth, age at last birth or number of children. The risk patterns were similar for localized and advanced breast cancer and the results are therefore not shown.

## Discussion

We found contrasting effects of length of birth interval on breast cancer between ductal and lobular cancers, and between younger (< 50) vs. older (50+) ductal cancers. Shorter intervals were associated with decreased risk for lobular cancers, while shorter intervals (especially between the 1st and 2nd, and 2nd and 3rd births) increased the risk of ductal cancer in premenopausal women. There was also indication of significantly higher risk for especially short intervals of less than one year for the premenopausal ductal cancers.

Previous breast cancer incidence models show that increasing spacing between the 1st and 2nd child birth increases the overall breast cancer risk [15, 17, 22]. Russo & Russo suggested that though the breast exhibits maximum differentiation after the first pregnancy, not all tissue might have been fully differentiated [23]. A close second pregnancy further differentiates breast cells and therefore offers less time to accumulate DNA damage [4, 24]. Pregnancy has a dual effect on breast cancer risk with a short-term increase in the risk followed by a long-term protective effect, prominently for first full term pregnancy [2, 4, 25]. Maximal cellular differentiation and maturation of the breast occurs after the first full-term pregnancy, making the breast cells more resistant to carcinogenic effects. Our finding that postmenopausal women showed no increase in risk of ductal and lobular breast cancer for short birth interval categories seems to confer to the long term protection due to terminal differentiation of mammary glands [23, 26]. Patients with lobular breast cancer appear to be almost always hormone receptor positive and thus specifically sensitive to hormonal factors [27, 28].

On contrary to the findings by Russo [26], the risk of premenopausal ductal breast cancer subtype in our study was higher when the birth interval between the 1st and 2nd birth was shorter and the reduced risk was observed for longer intervals. In closely occurring pregnancies, the mammary cells are repeatedly exposed to high amounts of estrogens and other steroids, which may be associated with the increased risk of ductal breast cancer in these women. Thus, our finding might suggest that for premenopausal breast cancer increased risk following pregnancy outweighs the long term protective effect. The long term protective effect due to pregnancies may not be reached before the age of 50 years.

In lobular breast cancer, the lowest ORs were virtually systematically observed in shortest birth intervals. Lobular breast cancer develops in the cells lining the lobules, which work as milk-producing elements. During many hundreds of past years, women in primitive life conditions without contraceptive aids conceived and gave birth several times, with short intervals between pregnancies. Consequently, they had to take care of their newborns through breastfeeding up until the next pregnancy; which was usually quite soon if life conditions were appropriate for ovulatory menstrual cycling. Thus, the lobular cells probably had to be active for milk formation in a regular rhythm, with an interval of just 1–2 years. The importance of this interval gets support from a study on Finnish mothers with ten or more childbirths which showed that the natural inter-pregnancy interval of 8.5 months plus the gestational period of 9 months’ results in the natural inter-birth interval of 17.5 months, which is in the middle of the interval length where we observed lowest ORs for lobular breast cancer [29].

Breastfeeding is considered to have a protective effect against breast cancer risk by aiding the differentiation of mammary epithelium in its terminal phase [30, 31]. The protection gained from breastfeeding could also be due to long-term endogenous hormonal change, i.e., decreased estrogen and increased prolactin levels, thus, inhibiting initiation and growth of breast-cancer cells [32–34]. Several studies have shown an inverse association between duration of breastfeeding and risk of premenopausal breast cancer [34–39]. Though information on breastfeeding is lacking in the current study, it could be hypothesized that GM women with short intervals between the 1st and 2nd birth might have breastfed their children for only a short time. It is possible that in premenopausal women, part of the increase in ductal breast cancer risk observed in connection with a short interval below one year between the 1st and 2nd birth is defective breast maturation owing to a lacking or short breastfeeding period, and possibly also a lack of prolactin effect.

We also calculated the effect of average interval between 1st and 5th birth as the predictor of breast cancer risk and observed the significant association with lobular breast cancer only. The result is similar to the results of an earlier Finnish study based on partially same cohort as the present study but with somewhat different analysis method [5]. We are no aware of any other studies showing results on average (or total) interval between the 1st and 5th birth. Our result clearly indicate that use of average interval does not show all the characteristics of the risk related to each of the separate birth intervals. E.g., the strong association between 1st birth interval and premenopausal ductal breast cancer in our study was not seen in the results based on average birth interval.

Our study has some limitations. The information on breastfeeding history of the women was not available. It should also be noted that many of the key estimates involved in the discussion of postmenopausal ductal and lobular breast cancer subtypes in our study have wide confidence intervals; hence, the inferences based on such estimates are tentative at the best. Moreover, our study is based only on GM women cohort. Women capable to give birth to five or more children may be different from other women in terms of their physiology etc. Therefore, the findings concerning e.g. the effect of interval between the 1st and 2nd birth seen in our study may be different from those seen among women with only 2 children. To our knowledge, there are no studies among GM women in other populations. Moreover, the interval category < 1 year in our study has small numbers of observations. We still chose to present the findings from this category because a birth interval of less than 1 year clearly means that the pregnancy has been abnormal and according to our a priori theory may therefore carry an exceptional risk of breast cancer.

The ages at first and last birth were not main study targets in this study but they were used for adjustment purposes. It is reassuring to see that we saw significantly increasing trends in risk of lobular breast cancer with increasing age at first birth and in risk of premenopausal ductal breast cancer with increasing age at last birth as expected based on earlier literature [40, 41].

## Conclusion

Our results indicate that two first pregnancies occurring with shorter intervals might increase the risk of ductal breast cancer among premenopausal women, whereas interval length may not be related to ductal breast cancer among postmenopausal grand multiparous women. For the lobular breast cancer, shorter intervals seem to be protective up to the fifth child. Our hypothesis was that the “natural birth interval” of 1–2 years would carry the lowest risk. This was not always the case, e.g., the risk of premenopausal ductal breast cancer was lower when the interval between the 1st and 2nd birth was longer. Results for the exceptionally short intervals of less than one year as a separate category in our analysis were inconsistent and had wide confidence intervals but gave a hint that it may be warranted to also look at this category in future studies. The mechanisms behind our observations of variation in risk of subcategories of breast cancer associated with birth intervals are still speculative. Further studies, including information on breastfeeding are needed to better understand these associations.

## Abbreviations

CI: Confidence Interval
DNA: Deoxyribonucleic acid
GM: Grand-Multiparous
ICD: International Classification of Diseases
NPR: National Population Register
OR: Odds Ratio
